# “Carpe Diem?”: Disjunction Effect of Incidental Affect on Intertemporal Choice

**DOI:** 10.3389/fpsyg.2021.782472

**Published:** 2021-12-10

**Authors:** Lei Zhou, Tong Zou, Lei Zhang, Jiao-Min Lin, Yang-Yang Zhang, Zhu-Yuan Liang

**Affiliations:** ^1^School of Management, Guangdong University of Technology, Guangzhou, China; ^2^Social, Cognitive and Affective Neuroscience Unit, Department of Cognition, Emotion, and Methods in Psychology, Faculty of Psychology, University of Vienna, Vienna, Austria; ^3^School of Psychology, Shaanxi Normal University, Xi’an, China; ^4^CAS Key Laboratory of Behavioral Science, Institute of Psychology, Beijing, China; ^5^Department of Psychology, University of Chinese Academy of Sciences, Beijing, China

**Keywords:** intertemporal choice, incidental affect, disjunctive effect, time perception, immediacy effect

## Abstract

Incidental affect has an important impact on intertemporal choice (IC). This research aimed to test how positive incidental affect influences IC and its underlying mechanisms. We assumed that positive incidental affect may have a disjunction effect on IC that includes or excludes immediate time. Moreover, we examined the role of time perception for the effect of affect on IC. In Study 1, after undergoing affect priming by video clips, participants completed the IC task using a multiple staircase paradigm. Using Hierarchical Bayesian Modeling, we estimated the discount rate parameter by distinguishing “immediate” and “non-immediate” conditions of IC. The participants’ time perception was also measured. In Study 2, apart from the choice preference of IC, we additionally investigated the differences in the participants’ attention to delay and reward attributes before decision making. The results of the two studies indicated that positive incidental affect leads to longer time perception (Study 1) and prior and more attention to the delay attribute of IC (Study 2), which leads individuals to prefer immediate options in the IC (Studies 1 and 2). Moreover, there is a disjunction effect of affect; in other words, the incidental affect did not influence IC excluding immediate time (Studies 1 and 2). This study improves our understanding of the disjunctive effect and its mechanism of inducing a positive incidental affect on IC and thus provides a new perspective on how related decision making can be improved.

## Introduction

Intertemporal choice (IC) refers to the individual trade-offs between costs and benefits at different time points and the corresponding judgments and choices (Frederick et al., 2002). Compared with larger, distant future rewards, people tend to favor smaller, nearer future rewards, thus, temporal discounting is generated (Read, 2004). When rewards occur immediately, individuals tend to discount future rewards more, showing an immediacy effect (Frederick et al., 2002; Weber et al., 2007).

Affect is a central driving force behind decision making, impacting it either positively or negatively. Previous studies have shown that affect influences intertemporal preferences by changing time perception (Ifcher and Zarghamee, 2011; Laube and van den Bos, 2020). According to the Hot/Cool-System hypothesis, there is a “cool cognitive system” and “hot emotional system,” where affect has a greater influence on preferences of immediate reward/loss (Metcalfe and Mischel, 1999; Hirsh et al., 2010). Therefore, affect may have a disjunction effect on IC that includes or excludes immediate time. The current study hypothesized that affect only influences choice preference when IC includes immediate time, and after removing it, its impact on IC weakens. In the current study, taking positive incidental affect as an example, we set different conditions of IC (including or excluding immediate time) to explore the influence of affect on IC and clarify the role of time perception in the effect of affect on IC.

In IC studies, individuals choose between the smaller-sooner (SS) and larger-later (LL) options. Decision makers place a disproportionate weight on immediate outcomes and would change their preference from a smaller monetary outcome to a larger one as both outcomes become more distant in time. This is the so-called immediacy effect. For example:

**Program 1.** *A. Now, gain $30; B. After 1 d, a gain of $31.*

**Program 2.** *A′. After 100 d, a gain of $30; B′. After 101 d, a gain of $31.*

People often prefer immediate option A in *Program 1*, but after extending the time for both options, people prefer delayed option B′ in *Program 2* (Kirby and Herrnstein, 1995).

The explanations for the immediacy effect differ among the decision models. *The hyperbolic time discounting model* (Mazur, 1987) assumes that the value of a delayed outcome can be described by an exponential function. Therefore, individuals discount the value of delay at a different rate, and the discount for immediate time is the greatest, leading to a preference for the SS option. Gradually, individuals’ discount rate per unit time decreases; therefore, they prefer LL options. However, according to *the perceived time-based model* (Zauberman et al., 2009), such time-variant preferences are due to the perception of delays. Individuals perceive time subjectively rather than objectively, and the relationship between an objective change of time and an individual’s subjective time perception conforms to the Weber-Fechner law. For example, the subjective time perception of 3 years may be 1.3 times longer than 1 year (rather than 3 times longer; Zauberman et al., 2009). Therefore, individuals’ discount rate per unit of subjective time in IC is constant, and the preference reversal in IC is due to the different time perceptions. Accordingly, in Programs 1 and 2, individuals’ subjective time discounting remains unchanged, but the difference in subjective time perception in Program 1 is greater than that in Program 2, thus leading to preference reversal.

The immediacy effect has been widely studied and found to be robust across different situations (Zhou et al., 2019). At the behavioral level, the immediacy effect is an important source of dynamic inconsistency in decision preference (Read et al., 1999); at the neural level, two separate systems are involved in IC: choice pairs that include immediate options will preferentially engage limbic structures compared with those that exclude immediate options. While regions of the lateral prefrontal cortex and posterior parietal cortex are engaged uniformly by intertemporal choices irrespective of delays (McClure et al., 2004). In sum, these studies show the particularities of *immediate time* in IC.

Research on affect and decision making has burgeoned over the last several decades (George and Dane, 2016). These studies mainly investigate the impact of affect based on its valence, time of occurrence, or source. Early research principally focused on the role of valence, that is, how general positive or negative affect, or specific affect, influences IC (Jiang and Sun, 2019). Recently, to comprehensively examine the effect of affect from different sources, researchers divided affect into two categories: integral and incidental (Lerner et al., 2015). Loewenstein and Lerner (2003) defined integral affect as the affective influences that result from evaluating the decision or judgmental target itself, while incidental affect includes all factors that elicit affect but are unrelated to the judgment target. For example, college students on the eve of graduation are faced with two options: “attending a party” or “writing a paper.” There are differences not only in the reward magnitude and time dimension but also in their emotional attributes, that is, happiness or frustration. This is an integral effect.

Information unrelated to the task drives incidental affect, which is widespread and has an important influence on behavior. People may inevitably be influenced by these factors (e.g., sunny weather, background music in shopping malls, news about favorite team wins, and traffic jams), and experience incidental effects of pleasure, excitement, or irritability. As background information, incidental affect can be easily ignored. According to the view of “mood-as-information,” people often consider affect as a source of information, and make mood-congruent judgments based on asking themselves “*How do I feel about it*?” (Schwarz and Clore, 2007). Moreover, they may mistakenly take an emotion-based reaction as a reaction to the decision target. For example, information unrelated to the decision (e.g., news about a favorite team win) will affect people’s subjective well-being ratings. For IC, incidental affect will impact the degree of individual impulsivity, positive behaviors such as saving, or negative behaviors such as addiction.

Research on the impact of incidental effect on IC varies; however, the results, particularly for positive affect, are still controversial. Early research revealed its benefits for IC: Pyone and Isen (2011) found that it promoted cognitive flexibility, fostered a higher level of thinking, and cultivated a more future-oriented view of time, which could lower the time discount rate and thereby facilitate participants’ preference for LL options (Wang and Liu, 2009; Ifcher and Zarghamee, 2011; Pyone and Isen, 2011). Contrastingly, recent studies have also shown that its negative impact causes a higher time discount rate, resulting in increased impulsivity (Norouzi, 2011; Kim and Zauberman, 2013; Lempert et al., 2017; Zhou et al., 2021). Similarly, Laube and van den Bos (2020) found that positive integral affect can also cause individuals to perceive the same objective future periods to be subjectively longer, leading people to prefer SS options. One possible reason for such an effect may be that positive affect causes people to shift their focus more on delay rather than rewards in IC; thus, they become less patient.

To study the impact of affect, few studies have distinguished the delay conditions of IC, that is, including or excluding immediate time. According to the Hot/Cool-System hypothesis (the cool, cognitive “know” and the hot, emotional “go” system), individuals’ processing of LL options is mainly driven by the cool system, while the preference for SS options is more likely to be influenced by the hot system (Metcalfe and Mischel, 1999). Similarly, mood management theory posits that individuals arrange internal and external stimulus conditions to minimize bad moods and maximize good moods (Zillmann, 1988a,b). Thus, for IC, when individuals experience stronger positive affect, they prefer immediate rewards, which could be helpful in maintaining their present positive mood (Norouzi, 2011). Correspondingly, most measures to control affect in daily life also involve the effect of immediate time. For example, the US government has set a waiting period law to prevent impulsive acts of gun violence (Giffords, 2021), and the Federal Trade Commission implements the Cooling-Off Rule to protect American consumers (Consumer Information, 2021). Overall, it can be speculated that affect may influence the strength of the immediacy effect in IC, and after removing the immediate time, the impact of affect would be weaker, suggesting a disjunction effect of the incidental affect on IC. Therefore, we postulate the following hypothesis:

H_1_: If IC includes immediate time, participants from the affect-positive group are more likely to prefer SS options when making IC compared to the affect-neutral group; when IC excludes immediate time, no significant difference in intertemporal preferences between participants from affect-positive and affect-neutral groups will be observed.

The mechanism underlying the disjunctive impact of affect on intertemporal choice may be related to time perception. The perceived time-based model of IC indicates that individuals’ choice preferences for IC reflect their subjective time perceptions (Zauberman et al., 2009). Kim and Zauberman (2009) set two model parameters for decision makers: the overall level of time contraction (α) and time sensitivity (β), to measure individuals’ subjective perception of the length of time and their sensitivity to anticipatory time horizons. Related studies found that when the subjective time estimates were accounted for, discount rates were constant but no longer decreased on most time horizons, as hypothesized by the hyperbolic models. These results imply that the nonlinear time discounting assumed by the hyperbolic discount model can be explained by differences in subjective time perception. By incorporating subjective time perception in IC models, simple and linear decision models can better explain individuals’ choice preferences.

Manipulating incidental affect can influence IC preferences by changing time perception. Relevant studies have shown that when assessing the time of future pleasant events, people overestimate waiting times, which triggers feelings of impatience and increases impulsivity. When assessing unpleasant events, future time perception is shortened (Geoffard and Luchini, 2010). This may be because it affects changes in time perception by influencing physiological activation and the allocation of attentional resources (Buhusi and Meck, 2006; Wang and He, 2020). That is, attributes of affect, such as source, valence, intensity, and motivation, could change time perception by influencing physiological activation and the allocation of attention resources (Buhusi and Meck, 2006; Gil and Droit-Volet, 2012). Laube and van den Bos (2020) found that integral affect influences impatient behavior because it leads distortions in time perception and thus alters the weighting of choice attributes. In their study, to control for the influence of time perception on decision making, researchers integrated subjective time estimates into a dual-parameter hyperbolic discounting model for IC. The results showed that the difference in time discount rate disappeared, indicating that the difference in time perception led to a change in decision-making preferences. As background information for decision making, manipulation of incidental affect could lead to changes in people’s perception and weighting of time, thereby influencing intertemporal preferences. Therefore, we postulate the following hypotheses:

H_2_: Participants in the affect-positive group perceived the same objective future durations to be subjectively longer than those in the affect-neutral group.

H_3_: There is no significant difference in the subjective time discount rate of participants in the affect-positive and affect-neutral groups.

H_4_: Compared with the affect-neutral group, participants from the affect-positive group focus prior and more to the delay rather than the reward attribute of intertemporal options.

The current research distinguished IC as including/excluding immediate time conditions, to reveal the disjunction effect of incidental affect on IC and explored the mechanism underlying time perception. In Study 1, we assessed choice behavior using a staircase IC task. To test the disjunction effect of incidental affect on IC, we subdivided the task conditions into including/excluding immediate time conditions. We used the hierarchical Bayesian modeling (HBM) method to fit participants’ responses from different time conditions to the hyperbolic discounting model and calculated the discount rate as the dependent variable. Moreover, to examine whether time perception was prolonged in the affect-positive group, we systematically compared time perception between the affect-positive and affect-neutral groups. Finally, we incorporated time perception into decision models and examined the role of time perception in the disjunctive effect of incidental affect on IC. In Study 2, we tested the robustness of the effects found in Study 1 and further explored whether affect led people to pay asymmetrical weighting to the delay and reward attributes of IC.

## Study 1

### Methods

#### Participants

GPower software was used to estimate the required sample size for this study. A power analysis indicated that a total of 54 participants were needed for a medium partial η^2^(0.25) when α = 0.05 for a power of 0.95 with two independent groups, using a repeated measures analysis of variance (ANOVA), within-between-subject interactions. The final sample consisted of 62 college students from universities in Guangzhou and Shenzhen, China (32 women, *M*_age_ = 20.66, *SD*_age_ = 0.19). All participants had normal or corrected-to-normal vision. Written informed consent was obtained from all the participants prior to participation. Participants received CNY 20 (CNY 1 ≈ 0.15) in cash for participation. To further incentivize cooperation, participants were informed that at the end of the experiment, one IC pair would be randomly selected from the participants’ question set and would receive an extra payment (5% of the reward, CNY 5–10) according to his or her choice in that question. For example, if a participant chose an option with a 15-day delay, then he or she would receive payment 15 days from the day of participation.

#### Materials and Procedure

Participants were randomly assigned to the affect-positive (*n* = 31) or affect-neutral group (*n* = 31). They completed the tasks in the experiment and the questionnaire in turn, as follows.

At the beginning of the experiment, we aimed to prime participants’ affect. The affective priming materials were 5 min video clips. The video of the affect-positive group included well-known variety show clips and a “father-son play” movie. The video of the affect-neutral group included a news interview about the weather and highway snow scenes. The variety show clips for the affect-positive group were determined from pre-experiments,^1^ and other materials were all derived from the standardized Asian cultural sentiment database^2^ developed by Deng et al. (2017). After watching the video, participants in the affect-positive group were asked to list 3–5 things that made them feel happy in their lives and describe one thing that makes them the happiest, whereas those in the affect-neutral group were asked to list 3–5 common things in life and describe t classroom scenario (Briñol et al., 2007).

After affect priming, participants completed the staircase IC task, making a series of choices between SS and LL options (Hardisty et al., 2013). The task contains two conditions: delay of SS option is *immediate* (today) or *non-immediate* (15 days in the future), and each condition included five blocks (corresponding to five different delays for the LL option). For the “immediate” condition, the delays for LL option time were 7, 15, 30, 80, or 140 days. In the “non-immediate” condition, the delays of SS and LL options were all increased by 15 more days than the “immediate” condition.

**FIGURE 1:**
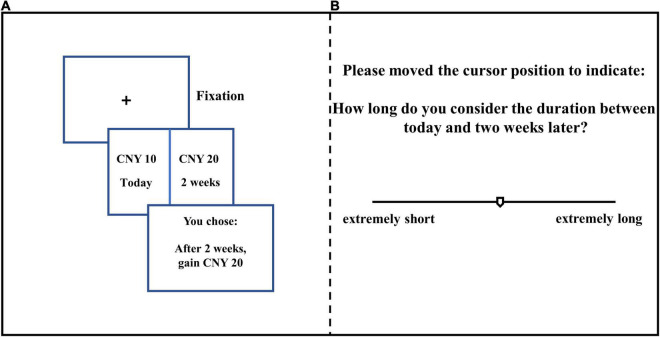
Experimental procedure with example tasks: **(A)** IC task, **(B)** Time perception task. [In both studies, the experimental materials were all presented in Chinese.] For A, the option pair is displayed until participants make a choice.

In each block, the reward of the LL option was fixed as CNY 200. The SS option began from CNY 100 and was adjusted through trials according to the participant’s choice. For example, the first presented option: (a) *15 days, CNY 200*; (b) *Today, CNY 100*. If the participant chose (a), the reward of the SS option presented for the second trial would be changed to the intermediate value of the LL and SS options from the first trial, that is, option (b) became (b’) *Today*, *CNY 150*. The range of SS options in the second trial reward was 100–200. However, if participants chose (b) in the first turn, the reward of the SS option in the second trial changed to an intermediate value of 0 and the SS option, that is, option (b) became (b’) *Today, CNY 50*. Here, the range of the SS option for the second trial reward was 0–100. The block ended when the range of the SS option reward is no more2+ than five. One practice block was first presented to familiarize the participants with the experimental procedure, and participants could have 1 min of rest between each block and at least 2 min of rest between each condition (see Figure 1A).

After the IC task, participants completed a subjective time perception measurement for all delays from the IC task using a non-numerable line scale (Zauberman et al., 2009). In detail, a non-numerable line segment with a length of about 28.5 cm was presented on a computer screen, with a movable cursor in the middle, “extremely short” label on the left end, and “extremely long” label at the right end (see Figure 1B). Participants moved the cursor position and set a line segment of a certain length to represent the length of the time horizon. Before starting, participants were informed that they would estimate the length of nine time periods within the interval of 7 to 160 days, and the order of each time period was random. Participants practiced before the experimental session.

Finally, participants completed the Chinese version of the Basic Positive Emotion subscale of the Positive and Negative Affect Schedule (PANAS; Guo and Gan, 2010), and the evaluation questionnaire of affect priming materials (Deng et al., 2017). In this measurement, participants evaluated the familiarity, likability, motivation, valence, and arousal of the affect priming materials on a nine-point scale (1 = extremely low level, 9 = extremely high level; see Supplementary Material).

### Results

#### Affect Priming

**FIGURE 2:**
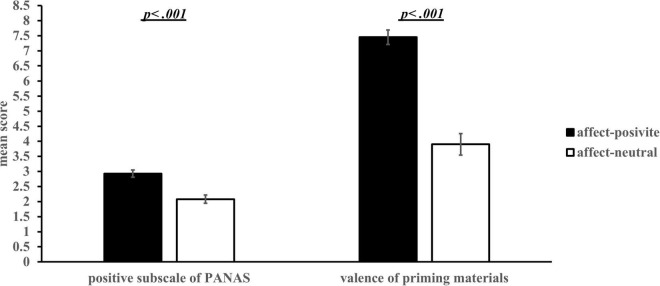
Mean scores of positive subscale of PANAS and the evaluation for the valence of priming materials in different affect groups *(M ± SE).* The item for valence evaluation was *“Did you feel happy when watching the video”* using a nine-point Likert scale (1 = very unhappy, 9 = very happy).

We tested the difference in the scores of the positive subscale of PANAS and the evaluation of priming materials between the affect-positive and affect-neutral groups. The positive affect score of PANAS in the affect-positive group was significantly higher than that in the affect-neutral group, *t*(60) = 4.65, *p* < 0.001, Cohen’s *d* = 1.18, 95% CI = [0.49, 1.23] (see Figure 2). For the evaluation of priming materials, there was no significant difference in the familiarity of the video between the affect-positive (*M* = 4.94, *SE* = 0.46) and the affect-neutral group (*M* = 4.03, *SE* = 46), *t*(60) = 1.39, *p* = 0.17, 95% CI = [–0.40, 2.20]. Compared with the affect-neutral group (*M* = 3.48, *SE* = 0.27), participants in the affect-positive group (*M* = 6.65, *SE* = 0.36) liked the video more, *t*(60) = 6.94, *p* < 0.001 Cohen’s *d* = 1.77, 95% CI = [2.25, 4.07], and experienced more pleasure, *t*(52.58) = 8.10, *p* < 0.001, Cohen’s *d* = 2.06, 95% CI = [2.67, 4.42], and amusement, *t*(60) = 5.68, *p* < 0.001, Cohen’s *d* = 1.44, 95% CI = [1.76, 3.66]. Meanwhile, participants in the affect-positive group (*M* = 6.65, *SE* = 0.36) had a significantly higher motivation to enter the video scene than those in the affect-neutral group (*M* = 3.48, *SE* = 0.27), *t*(60) = 4.47, *p* < 0.001, Cohen’s *d* = 9.96, 95% CI = [1.19, 3.12]. Overall, the results indicate that affect priming was effective, and the affect-positive group experienced a significantly higher degree of pleasure than the affect-neutral group.

#### Objective Time Discounting

We fitted a hyperbolic discounting model (among other candidate models) to participants’ choice data to obtain their discount rate, using the HBM method. The analysis was performed using the R software package hBayesDM (hierarchical Bayesian modeling of decision-making task; Ahn et al., 2017; Zhang et al., 2020). All model fittings used four independent Markov chain Monte Carlo (MCMC) chains, and each chain contained 1,000 valid samples after initial tunning of the MCMC algorithm. Therefore, the distribution of all parameters consisted of 4,000 valid posterior samples. A Gelman-Rubin test (Gelman and Rubin, 1992) showed that the $\hat{R}$ of all parameters was less than 1.1, indicating that the four independent MCMCs had converged, and the results of model fitting were stable and reliable.

First, referring to the common method of previous studies, the data were fitted without distinguishing between “immediate” and “non-immediate” conditions. Next, we distinguished the two time conditions to fit the data again. Finally, we used widely applicable information criterion (WAIC; Vehtari et al., 2016) to compare the models obtained by two different fitting methods. The WAIC uses all MCMC posterior samples to calculate the out-of-sample predictive accuracy of the model. To avoid overfitting, it penalizes the model complexity (see Supplementary Material for WAIC calculation formulas). A lower WAIC score indicates a better out-of-sample prediction accuracy of the candidate model. If ΔWAIC > 10, the model is considered to be significantly different (Burnham and Anderson, 2004).

The results showed that when the model was fitted by distinguishing “immediate” and “non-immediate” conditions, whether in the affect-positive (WAIC = 3220) or affect-neutral group (WAIC = 3281), the WAIC was significantly lower than models that jointly fitted data from the “immediate” and “non-immediate” conditions (affect-positive group: WAIC = 2311, ΔWAIC = 909; affect-neutral group: WAIC = 2285, ΔWAIC = 996). The results showed that the experimental data can be better fitted, and a better model can be obtained after distinguishing “immediate” and “non-immediate” conditions. Thus, we adopted a decision model that distinguished the time conditions for further analysis.

**FIGURE 3:**
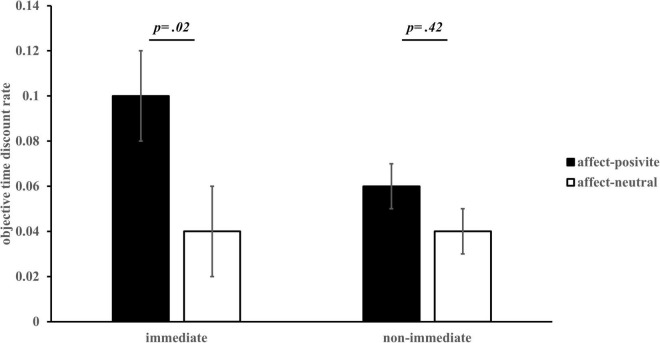
Objective time discount rate for different affect groups *(M ± SE)*.

Taking the discount rate estimated above as the dependent variable, 2 (affect) × 2 (task conditions) analysis of variance (ANOVA) showed that the main effect of affect was significant, *F*(1, 60) = 3.34, *p* = 0.07, η^2^ = 0.05, 95% CI = [−0.004, 0.08]. The time discount rate of the affect-positive group (*M* = 0.08, *SE* = 0.02) was larger than that of the affect-neutral group (*M* = 0.04, *SE* = 0.02). The main effect for task conditions was significant, *F*(1, 60) = 4.81, *p* = 0.03, η^2^ = 0.07, 95% CI = [0.002, 0.03]. The discount rate of the “immediate” condition (*M* = 0.07, *SE* = 0.01) was significantly greater than that of the “non-immediate” condition (*M* = 0.05, *SE* = 0.01), indicating an immediacy effect. The interaction between affect and task conditions was significant, *F*(1, 60) = 8.84, *p* = 0.004, η^2^ = 0.13. Bonferroni adjustments were used for post-hoc pairwise comparisons. Post-hoc analysis showed that in the condition of “immediate,” the time discount rate of the affect-positive group was significantly larger than that of the affect-neutral group, *p* = 0.02, 95% CI = [0.01, 0.12]; in the condition of “non-immediate,” the time discount rate of the affect-positive group was not significantly different from that of the affect-neutral group, *p* = 0.42 (see Figure 3).

Overall, these results support the hypothesis that the influence of incidental affect on IC has a disjunction effect; when including “immediate time,” positive incidental affect causes individuals to prefer smaller-sooner options in IC, and the strength of immediacy effect is stronger when IC excludes “immediate time.” Positive incidental affect has no effect on choice preferences (Hypothesis 1).

#### Time Perception

We calculated and compared the subjective time perceptions of the two affect groups (Kim and Zauberman, 2009). In detail, the measured length of the line scale was transformed into day units by setting the overall mean distance for the one-week duration as the baseline unit (e.g., *M*_one week_ = 7.42 cm; 7.42 cm represents the subjective time of a week) for the time judgment by each individual. Then, the subjective time perception “T” for each delay was calculated. Furthermore, in order to compare different lengths of time perception from a unified dimension and examine the overall level and sensitivity of time perception, a power function model of time perception was used to fit their time perception data into “immediate” and “non-immediate” conditions and estimate the time perception model parameters of each participant (Kim and Zauberman, 2009; Cruz Rambaud and Ventre, 2017; Cruz Rambaud et al., 2018; Laube and van den Bos, 2020)^3^ :


(1) $$T=\alpha t^{\beta_{3}}$$

where α is a scaling parameter that captures the overall degree of time contraction (how long or short individuals perceive time to be overall), and β is a nonlinear scaling parameter representing diminishing sensitivity to time.

**FIGURE 4:**
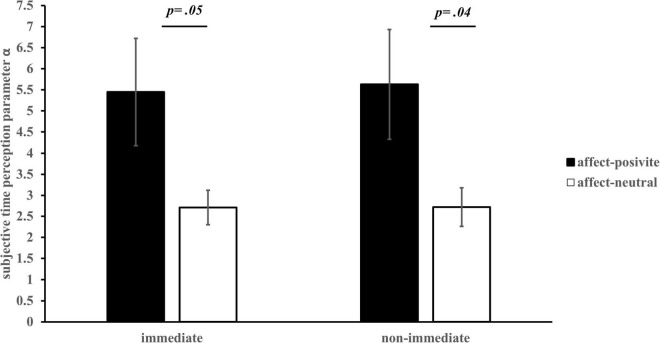
Subjective time perception parameter α in different affect groups *(M ± SE)*.

The results of a 2 (affect) × 2 (task conditions) ANOVA revealed that, for the overall perception of time (α parameter), the main effect of affect was significant, *F*(1, 60) = 4.36, *p* = 0.04, 95% CI = [0.12, 5.52]). However, neither the main effect of task conditions, *F*(1, 60) = 0.31, *p* = 0.58, nor the interaction between the two, *F*(1, 60) = 0.22, *p* = 0.64 (see Figure 4) were significant. For sensitivity to time (β parameter), none of the main effects of affect, task conditions, and their interaction were significant, affect: *F*(1, 60) = 2.17, *p* = 0.15; task conditions: *F*(1, 60) = 0.48, *p* = 0.49; interaction: *F*(1, 60) = 0.004, *p* = 0.95. These results support the hypothesis that compared with the affect-neutral group, participants in the affect-positive group had a longer subjective perception of time, but there was no difference in the time sensitivity between the two conditions (Hypothesis 2).

#### Subjective Time Discounting

To control for the impact of time perception differences on decision-making results, we integrated the subjective time estimate into the intertemporal decision model and replaced the time parameter *t* in the intertemporal decision model with the corresponding subjective time Perception *T* and estimated and compared the model parameters of the different participant conditions (Laube and van den Bos, 2020).

**FIGURE 5:**
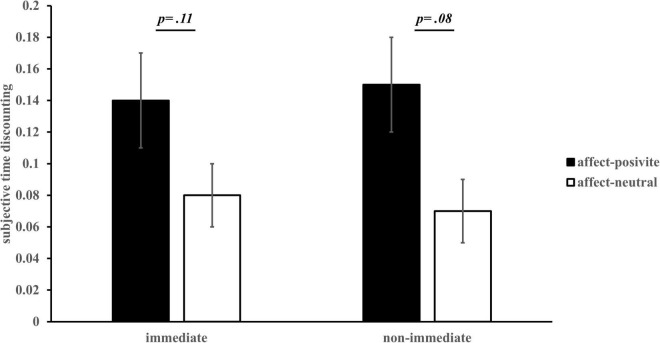
Subjective time discount rate in different groups *(M ± SE)*.

Results of a 2 (affect) × 2 (task conditions) ANOVA showed that the main effect of affect was marginally significant, *F*(1,60) = 3.03, *p* = 0.09, 95% CI = [–0.01, 0.14]. However, neither the main effect of conditions, *F*(1, 60) = 1.27, *p* = 0.27, nor the interaction between the two were significant, *F*(1, 60) = 2.24, *p* = 0.14 (see Figure 5). The above results support the hypothesis that after integrating subjective time perception into the IC model, no differences were found in discount rates, indicating that the subjective difference in time perception impacted choice preference in IC (Hypothesis 3).

In sum, Study 1 revealed the disjunctive effect of incidental affect on IC and the underlying mechanism of time perception. After priming positive incidental affect, individuals perceived time as a longer and preferred the SS option when making choices of IC, including immediate time. Incidental affect has no impact on individuals’ choice preferences for IC, excluding immediate time.

## Study 2

Study 1 showed the disjunctive effect of incidental affect on IC, and time perception was an important mechanism. However, we did not examine how people processed the delay and reward attributes of IC after priming positive affect. Therefore, in order to further clarify the mechanism of effect on IC, we adopted an attribute attention test to investigate whether incidental affect led to asymmetrical attention toward the delay and reward attributes of IC.

### Methods

#### Participants

A power analysis indicated that a total of 118 participants were needed for a medium partial η^2^(0.4) when α = 0.05 for a power of 0.95, using a χ^2^ test. The final sample consisted of 120 college students from a university in Guangzhou, China (59 women, *M*_age_ = 20.2, *SD*_age_ = 7.49). All participants had normal or corrected-to-normal vision. Written informed consent was obtained from all the participants prior to participation. They received credit for their course grades and CNY 5 payments for participating.

#### Materials and Procedure

Participants were randomly assigned to affect-positive (*n* = 60) or affect-neutral (*n* = 60) groups. They completed the experimental tasks and the questionnaire. At the beginning of the experiment, participants were primed with affect, as in Study 1.

After affect priming, participants completed the attribute attention test. The purpose of this test was to investigate whether the primed positive affect led to differences in individuals’ attention weight to reward and delay attributes in IC (Laube and van den Bos, 2020). The experiment was designed according to the principles of query theory (Weber et al., 2007). First, participants were introduced to the meaning of IC through examples and an attribute combination of each option (SS option: “shorter delay,” “smaller reward“; LL option: “longer delay,” “larger reward“) and completed a question to test whether they understood the meaning of each attribute as a manipulation check. Next, participants were presented with a set of IC options; however, the attributes of the options were all obscured, and there were only four boxes marked with “shorter delay,” “longer delay,” “smaller reward,” and “larger reward.” Participants could open a box to acquire information to make a choice. There were two conditions in this test: the “full choice condition” and “constrained choice condition.” In the “full choice condition,” participants could choose to open all the boxes in turn to acquire information about all attributes of a choice to investigate which attribute participants would look for first. In the “constrained choice condition,” participants could select only three of the four pieces of information to investigate which attribute participants to focus on more (i.e., “two delay attributes, one reward attribute” or “two reward attributes, one delay attribute“; see Supplementary Material for the experiment materials).

The participants then performed the IC task. The experimental material was obtained by simplifying it from Study 1. The task contains two conditions: the delay of the SS option is *immediate* (today) or *non-immediate* (15 days in the future), and each condition included 10 trials (corresponding to 10 different rewards for the SS option). For the “immediate” condition, the delay for the LL option time was fixed as 85 days in the future, and in the “non-immediate” condition, the delay of the LL option was fixed as 105 days in the future. The reward of the LL option was fixed as CNY 200, and the SS option for each condition ranged from CNY 100 to CNY 194. Finally, the participants completed the same questionnaire as in Study 1.

### Results

#### Manipulation Check

Five participants did not pass the manipulation check for the attribute attention task and were excluded from further analysis. Therefore, the final number of people included in the analysis was 115 (*N*_*positive*_ = 57, *N*_neutral_ = 58).

#### Affect Priming

**FIGURE 6:**
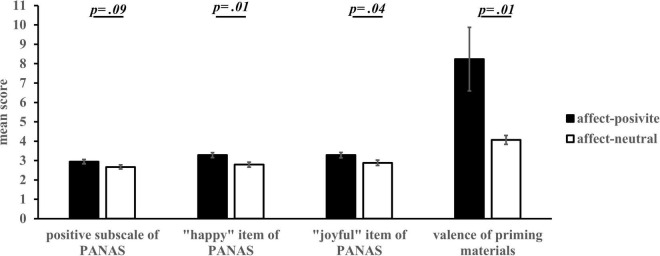
Scores of positive subscale of PANAS and the evaluation of priming materials in different affect groups *(M ± SE)*.

The test results of the difference in the PANAS positive subscale scores between the affect-positive and affect-neutral groups showed that the positive affect score of PANAS in the affect-positive group was marginally significantly higher than that in the affect-neutral group, *t*(113) = 1.69, *p* = 0.09, 95% CI = [−0.05, 0.59] (see Figure 6). The results of analysis of the five subscale items showed that there were no significant differences between the two conditions in the dimensions of “cheerful,” “delighted,” “excited,” “lively,” and “enthusiastic” (0.24 < *p* < 0.58); however, the “happy” and “joyful” degrees of the affect-positive group were significantly higher than those of he affect-neutral group, “happy”: *t*(113) = 2.59, *p* = 0.01, Cohen’s *d* = 0.48, 95% CI = [0.11, 0.86]; “joyful”: *t*(113) = 2.04, *p* = 0.04, Cohen’s *d* = 0.38, 95% CI = [0.01, 0.79].

For the evaluation of the priming materials, no significant difference in the familiarity of the video between the two groups was observed (*M*_positive_ = 5.39, *SE*_positive_ = 1.71; *M*_neutral_ = 3.97, *SE*_neutral_ = 0.29), *t*(113) = 0.83, *p* = 0.41, Cohen’s *d* = 0.15, 95% CI = [−1.99, 4.83]. Compared with the affect-neutral group (*M* = 3.84, *SE* = 0.23), participants from the affect-positive group (*M* = 7.47, *SE* = 1.66) liked the video more, *t*(113) = 2.18, *p* = 0.03, Cohen’s *d* = 0.41, 95% CI = [0.34, 6.92], and experienced more pleasure, *t*(113) = 2.53, *p* = 0.01, Cohen’s *d* = 0.47, 95% CI = [0.90, 7.42], and amusement, *t*(113) = 1.96, *p* = 0.05, Cohen’s *d* = 0.37, 95% CI = [−0.03, 6.63]. Meanwhile, participants in the affect-positive group (*M* = 7.79, *SE* = 1.66) had a significantly higher motivation to enter the video scene than the affect-neutral group (*M* = 4.60, *SE* = 0.27), *t*(113) = 1.92, *p* = 0.06, Cohen’s *d* = 0.36, 95% CI = [-0.11, 6.48]. Overall, the results indicate that affect priming was effective, and the affect-positive group experienced a significantly higher degree of pleasure than the affect-neutral group.

#### Intertemporal Choice

**FIGURE 7:**
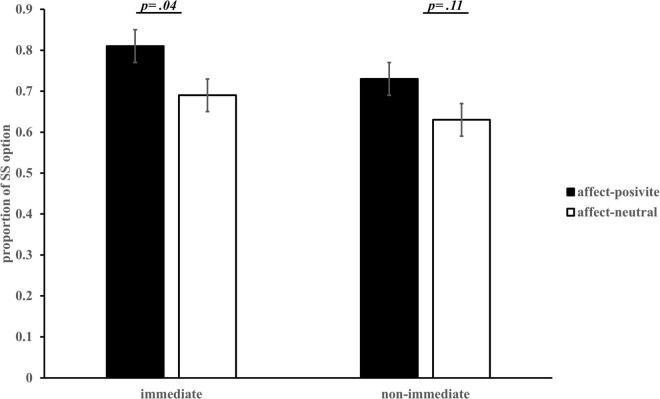
Proportion of SS option in different groups *(M ± SE)*.

Taking the proportion of SS option as a dependent variable (Zhou et al., 2019), a 2 (affect) × 2 (task conditions) ANOVA showed that the main effect of affect was marginally significant, *F*(1, 113) = 3.68, *p* = 0.06, 95% CI = [−0.004, 0.22]. Participants in the affect-positive group (*M* = 0.77, *SE* = 0.04) preferred the SS option more than those in the affect-neutral group (*M* = 0.66, *SE* = 0.04). The main effect of task condition was significant, *F*(1, 113) = 10.80, *p* = 0.001, η^2^ = 0.09, 95% CI = [0.03, 0.10]. The proportion of SS options in the “immediate” condition (*M* = 0.75, *SE* = 0.03) was significantly higher than that in the “non-immediate” condition (*M* = 0.68, *SE* = 0.03), indicating an immediacy effect. Their interaction was not significant, *F*(1, 113) = 0.22, *p* = 0.64. The Bonferroni post-hoc analysis showed that in the “immediate” condition, the proportion of choosing SS options in the affect-positive group was significantly higher than that in the affect-neutral group, *p* = 0.04, η^2^ = 0.04, 95% CI = [0.003, 0.24]. In the “non-immediate” condition, there was no significant difference between the affect-positive and affect-neutral groups (*p* = 0.11; see Figure 7).

Overall, these results replicate the main findings of Study 1 and support our hypothesis (Hypothesis 1), indicating that incidental affect has a disjunction effect on IC.

#### Attention Toward Attributes

**FIGURE 8:**
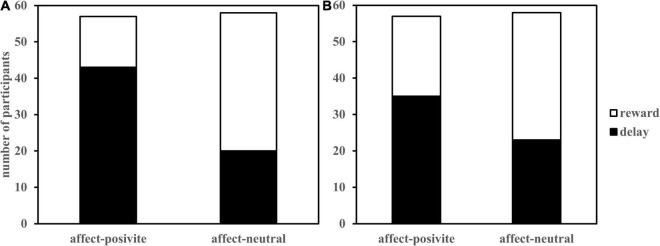
Attributes that participants pay attention to in attributes attention test: **(A)** attribute of first attention, **(B)** attribute of most attention.

In the “full choice condition,” participants could choose to open all the boxes to acquire information about all attributes. The results showed that participants were more likely to first open the box representing the delay attribute (“shorter delay” and “longer delay”) in the affect-positive group than the affect-neutral group, χ^2^ (1) = 19.47, *p* < 0.001, *r* = 0.41 (see Figure 8A). In the “constrained choice condition,” participants could only choose “two delay attributes, one reward attribute” or “two reward attributes, one delay attribute.” The results indicated that participants in the affect-positive group were likely to open more boxes representing the delay attribute than those in the affect-neutral group, χ^2^ (1) = 5.44, *p* = 0.02, *r* = 0.22 (see Figure 8B). The results indicate that compared with the affect-neutral group, the affect-positive group prioritized and paid the most attention to the delay attribute of IC, rather than the reward attribute.

Altogether, these results support the hypothesis that affect can lead to differences in processing IC in terms of delay and reward attribute. Compared with the affect-neutral group, after priming positive affect, individuals will prioritize and pay more attention to the delay attribute before making an IC (Hypothesis 4).

## Discussion

The current research revealed the disjunction effect of incidental affect in IC; it also revealed that a shift in subjective time perception is the underlying mechanism of this effect. Across two studies, we consistently found that positive incidental affect was associated with an increased impatient decision for IC that includes immediate time. However, no change in choice preference for IC that excludes immediate time was observed. Moreover, positive incidental affect changes intertemporal preferences by causing the individual to perceive the same objective future durations to be subjectively longer. Before making a decision, individuals paid more attention to time and were more inclined to know about the delay of intertemporal options, rather than reward attributes.

### Disjunction Effect of Affect on Intertemporal Choice

The finding that positive incidental affect can cause individuals to show stronger an immediacy effect in IC is consistent with previous studies (Norouzi, 2011; Kim and Zauberman, 2013; Lempert et al., 2017). These results are also consistent with the findings of Laube and van den Bos (2020), which indicated that integral affect leads to increased impatience in intertemporal choice. However, in contrast to these studies, using HBM, we distinguished IC including/excluding immediate time when estimating model parameters and obtaining a better model to reveal the disjunction effect of incidental affect. Studies 1 and 2 consistently showed that affect only influences IC, which includes immediate time, while individuals’ choice preferences remain the same when excluding immediate time.

Our finding, however, is different from that of other studies, which found that positive affect corresponds to long-term preferences for IC (Wang and Liu, 2009; Ifcher and Zarghamee, 2011; Pyone and Isen, 2011). Considering the difference in evaluation of affect materials between affect-positive and neutral groups, this divergence of influence on IC may be related to the fact that although the valence of the emotional priming materials is identical, there were differences in other dimensions, such as emotional intensity, self-involvement, and motivation. This speculation is supported by Lempert et al. (2017), who showed that when imagining positive experiences and recalling positive memories, although both can prime positive affect, they have opposite effects on the degree of individual impulsivity. Therefore, incidental affect has a complicated influence on decision making. Future research should focus not only on common dimensions, such as valence and arousal, but also on other dimensions of affect, such as motivation and involvement.

### Mechanisms Underlying the Impact of Incidental Affect in Impatient Choice: Time Perception

Similar to the findings of integral affect and other related studies (Geoffard and Luchini, 2010; Laube and van den Bos, 2020), the current research also found that after the priming of positive affect, individuals subjective time perception was longer. Moreover, in Study 2, we also revealed that incidental affect will also lead individuals to pay more attention to the delay attribute of IC. These studies showed that positive affect influences intertemporal preferences by making individuals overestimate time and increasing their attention to time. Overall, the above studies consistently demonstrate that time perception is the mechanism by which it influences IC; when an individual has a long-term perception, the waiting time could be perceived as a cost, which reduces the attractiveness of future options and causes higher impulsivity in IC (Wittmann et al., 2007; Wittmann and Paulus, 2008; Norouzi, 2011; Kim and Zauberman, 2013).

According to the assumption of the classic time discounting models, compared with the immediate rewards, distant rewards are afforded a smaller weight (Read, 2004). According to these models, time interval is like a “ruler,” and its length determines the degree which the individual will lose value in the future. The scale of this “ruler” is not uniform; thus, the discount rate varies at different time intervals. According to the hypothesis of the perceived time-based model, changing the delay time of IC leads to the reversal of decision preferences, not a change in discount rate, but a change in subjective time perception (Kim and Zauberman, 2009). Therefore, when people weigh options that occur at different times, the “ruler” they use is highly dependent on individual differences in their time judgments. Combined with the results of this research, factors such as affect can change behavioral decision making by influencing subjective time perception. It should be noted that in Study 1, there was no difference in the subjective time discounting between IC conditions (including/excluding immediate time), suggesting that compared with the results of objective time discounting, after controlling for the impact of time perception on decision-making results, the immediacy effect disappeared. This result indicated that time perception is an important mechanism for affect in IC, and future studies are necessary to examine whether it has such a consistent and strong role in other behavioral effects of IC.

### Implications

Theoretically, this research distinguishes the time conditions of IC and reveals the disjunction effect of incidental affect on IC. That is, incidental affect only influences information about immediate time of IC. Computationally, using HBM, we estimated the discount rate parameter by distinguishing “immediate” and “non-immediate” conditions of IC and obtained a better model than that jointly fitting data from different conditions. Moreover, the research reveals that incidental affect leads to differences in levels of individual attention paid to the delay and reward attributes of IC. Based on the perceived time-based model, through the test of subjective time discounting, this research shows that affect influences intertemporal preferences by changing time perception. In sum, this research provides more accurate answers to how incidental affect impacts IC.

This research also provides a new perspective on how to improve related decision-making behaviors. Based on the findings, positive incidental affect would lead to a stronger immediacy effect by influencing time perception. In the investment field of China, the sales of financial products have a “24-hour cooling-off period” rule, that is, consumers have the right to unilaterally and unconditionally cancel the contract within a reasonable time (China Banking and Insurance Regulatory Commission, 2019). This rule is consistent with our findings. Thus, from the perspective of regulators, shielding immediate time in IC can reduce consumers’ irrational decision-making behavior and protect their rights; from the perspective of consumers or decision makers, recognizing the important relationship between affect and the immediacy effect can reduce short-sighted impulsive behaviors in life and improve IC.

### Limitations and Perspective

First, this research is based on incidental affect, distinguishing only according to emotional valence and comparing positive and neutral affect. However, according to the results of Study 1, there are differences in the dimensions of excitement and motivation between the two conditions. Combined with other studies that examine the effect of positive affect on IC (Wang and Liu, 2009; Ifcher and Zarghamee, 2011; Pyone and Isen, 2011), and finding that imagining positive experiences and recalling positive memories have differential effects on behavior (Lempert et al., 2017), we can infer that even with the same valence, differences in emotional strength, self-involvement, motivation, and other dimensions will have differential effects on behavior. In addition to distinguishing affect according to common dimensions such as salience, valence and arousal, future related research should also investigate other dimensions of affect, such as motivation and involvement. In addition, according to the two-system analysis, which can be applied to positive and negative affect, negative affect may be similar to positive affect, which influences the immediacy effect of IC by affecting time perception. Future research can investigate the mechanism and boundary effect of negative affect in order to make useful attempts to resolve related research disputes.

Additionally, this research only adopted behavioral experiments and model fitting methods and did not directly verify the influence mechanism of affect from the perspective of the decision-making process. According to the assumptions of the attribute-wise model of IC, an individual’s impulse level stems from differences in the weighting of delay and reward attributes (Dai and Busemeyer, 2014). Similarly, our research also finds that after decision makers are stimulated with positive affect, they pay more attention to delay attributes and hope to learn more about time before making a decision. Future research may utilize process tracing methods, such as eye tracking and mouse tracing, to directly test the attentional mechanisms underlying incidental affect on decision making. It would also aid in investigating the neural mechanisms facilitating the impact of affect on IC utilizing model-based neuroimaging.

## Conclusion

This research explores the disjunction effect of incidental affect on IC under different time conditions and the mechanism underlying time perception. It found that positive affect makes individuals pay more attention to the delay attribute of IC before decision making, perceive subjective time as longer, and lead to a stronger immediacy effect in IC.

## Data Availability Statement

The raw data supporting the conclusions of this article will be made available by the authors, without undue reservation.

## Ethics Statement

The studies involving human participants were reviewed and approved by Institutional Review Board (IRB) Committees at the Chinese Academy of Sciences. The patients/participants provided their written informed consent to participate in this study.

## Author Contributions

LZO: conceptualization, methodology, software, validation, formal analysis, data curation, writing—original draft preparation, review, and editing, supervision, project administration, funding acquisition. TZ: formal analysis, investigation, software, visualization, writing—original draft preparation, review, and editing. LZA: formal analysis, writing—review and editing. J-ML: writing—review and editing. Y-YZ: funding acquisition, writing—review and editing. Z-YL: conceptualization, methodology, validation, resources, data curation, writing—original draft preparation, review, and editing, supervision, project administration, funding acquisition. All authors contributed to the article and approved the submitted version.

## Conflict of Interest

The authors declare that the research was conducted in the absence of any commercial or financial relationships that could be construed as a potential conflict of interest.

## Publisher’s Note

All claims expressed in this article are solely those of the authors and do not necessarily represent those of their affiliated organizations, or those of the publisher, the editors and the reviewers. Any product that may be evaluated in this article, or claim that may be made by its manufacturer, is not guaranteed or endorsed by the publisher.

## Footnotes

1 In the pre-experiment, we first asked university students from where the experiment was conducted to recommend five videos that they thought most evoke positive emotions. The 20 students were randomly selected from the school to rate the emotional valence of these five videos. The variety show segment that evoked the most positive affect was used as priming material.

2 The movie clips were all selected from the emotional materials of the “happy” and “neutral” dimensions of the emotion database established by Deng et al. (2017).

3 We modeled the data using Steven’s power law and Weber-Fechner law, and used Bayesian Information Criterion (BIC) to measure the performance of two models. The results indicated that the power function (Steven’s power law) fitted the data better than Weber-Fechner law (BIC_Steven’s power law_ = 3960, BIC_Weber–Fechner law_ = 4142, a lower BIC score indicates a better fitting of data). We therefore used the power model in all subsequent analyses.
