# Supplementary material for: “Carpe Diem?”: Disjunction Effect of Incidental Affect on Intertemporal Choice
Source: Front Psychol. 2021 Dec 10;12:782472. doi: 10.3389/fpsyg.2021.782472 (PMC8702439; doi:10.3389/fpsyg.2021.782472)
Supplement: Supplementary file 1 [file Data_Sheet_1.docx]

Supplementary Material

# Study 1 Evaluation questionnaire of emotional priming materials^[[1]](#footnote-1)^

Instruction: Please recall the video you just watched and rate this video on the following five dimensions. The scoring range is 1-9, where 1 represents an extremely low level, 5 represents an intermediate level, and 9 represents an extremely high level. Please circle the corresponding numbers according to your comments.

| Familiarity: Familiarity with the video | not at all 1 2 3 4 5 6 7 8 9 very much |
| --- | --- |
| Likability: How much do you like the video | not at all 1 2 3 4 5 6 7 8 9 very much |
| Motivation: Want to approach or withdraw from the scene presented in the video | high desire to withdraw 1 2 3 4 5 6 7 8 9 high desire to approach |
| Valence: Did you feel happy when watching the video | very unhappy 1 2 3 4 5 6 7 8 9 very happy |
| Arousal: Intensity of the emotional reaction when watching the video | very calm 1 2 3 4 5 6 7 8 9 very aroused |

# Study 1 WAIC calculation formulas

$WAIC=-2(\hat{\mathrm{lpd}}-\hat{p}_{\mathrm{waic}})$ (2)

$\hat{\mathrm{lpd}}=\sum_{i=1}^{n} \log(\frac{1}{S}\sum_{s=1}^{s} p\left( y_{i} | \theta^{s} \right))$ (3)

$\hat{p}_{\mathrm{waic}}=\sum_{i=1}^{n} \mathrm{Var}_{s=1}^{S}(log(p\left( y_{i} | \theta^{s} \right)))$ (4)

where $\hat{\mathrm{lpd}}$ is the computed log pointwise predictive density, corresponding to the goodness-of-fit, $\hat{p}_{\mathrm{waic}}$ is the estimated effective number of parameters, corresponding to the complexity of the model.

# Study 2 Attributes attention test

Instruction: Please read the following text carefully and then answer the questions in 3.1-3.2, according to your real thoughts.

You may encounter a type of multiple-choice question in your life, which requires you to choose between the two options **"occurring at different times, different amounts/outcome"** For example:

1. *Today, I can get CNY 100. Though after seven days, I could get CNY 200.*

The above two options both mean **"you can get a certain amount of money at a certain time"**; both options include a **"delay attribute"** and **"reward attribute"** reward attribute’.

**Delay attribute:** The time **"today"** in option A is a **"shorter delay"**

The time **"after 7 days"** in option B is a **"longer delay"**

**Reward attribute:** The amount **"CNY 100"** in option A is a **"smaller reward"**

The amount **"CNY 200"** in option B is a **"larger reward"**

3.1 **C.** "After 1 month, I will get CNY 50"; **D.** "After 1 week, I will get CNY 35." Among options C and D, which option has the "shorter delay" attribute? ( ).

3.2 The four boxes in the figure below are marked with "shorter delay," "longer delay," "smaller reward," and "larger reward." When you open the box, information can be acquired concerning all the attributes of a choice.


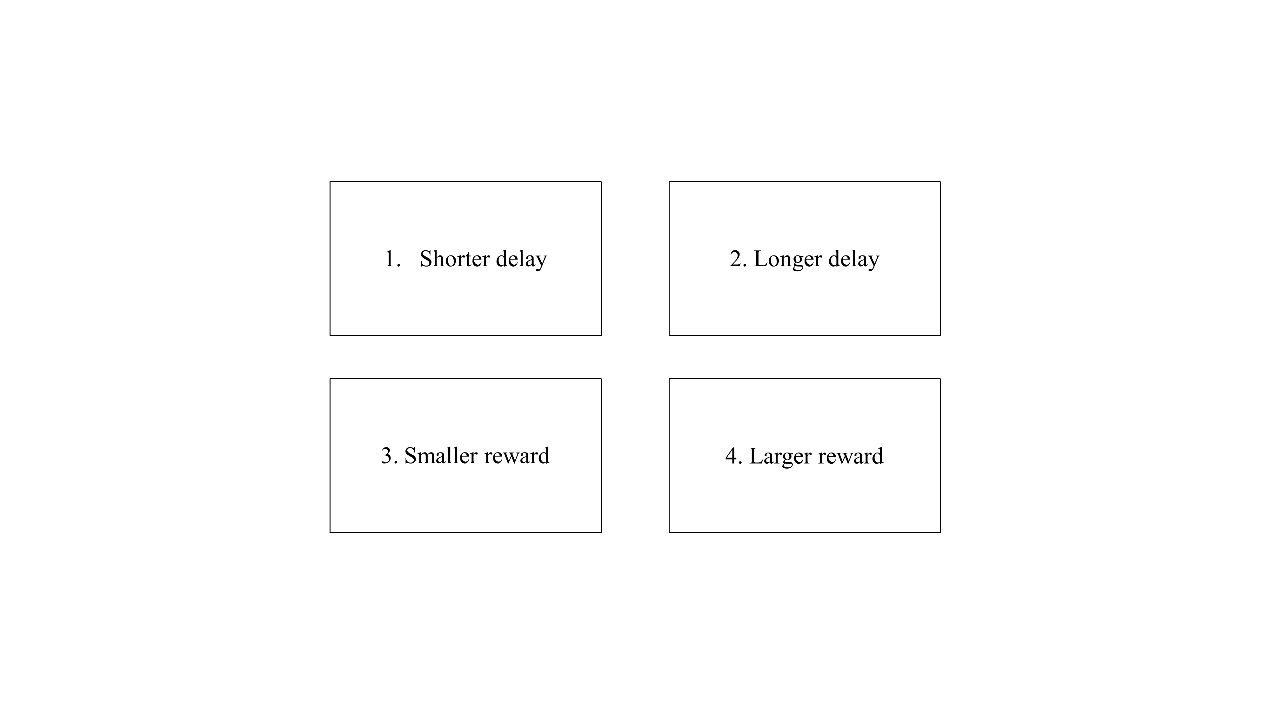


3.2.1 If you can open all the boxes above to acquire information from the boxes to make a choice. Please write down the order in which you would open the boxes below (Please write down the box numbers).

3.2.2 If you can only open three of the above three boxes to acquire information from the boxes to make a choice. Please write down the three boxes you chose to open. (Please write the box numbers).

1. In both studies, the questionnaire and instructions were all presented in Chinese. [↑](#footnote-ref-1)
